# COVID-19 Vaccination and Transient Increase in CD4/CD8 Cell Counts in People with HIV: Evidence from China

**DOI:** 10.3390/vaccines12121365

**Published:** 2024-12-03

**Authors:** Yanyan Li, Yingying Lin, Yunyun Yi, Na Zhu, Xinyu Cui, Xin Li

**Affiliations:** 1 Center of Integrative Medicine, Beijing Ditan Hospital, Capital Medical University, Beijing 100015, China; 15736990162@163.com (Y.L.); zhuna202203@163.com (N.Z.); cxy13373145868@163.com (X.C.); 2 National Center for Infectious Diseases, Beijing Ditan Hospital, Capital Medical University, Beijing 100015, China; 3 Center of Integrative Medicine, Peking University Ditan Teaching Hospital, Beijing 100015, China; linyingying0123@163.com; 4 Tuberculosis Prevention and Control Key Laboratory, Beijing Key Laboratory of New Techniques of Tuberculosis Diagnosis and Treatment, Senior Department of Tuberculosis, The Eighth Medical Center of PLA General Hospital, PLA General Hospital, Beijing 100853, China; yyygrace@126.com

**Keywords:** HIV, COVID-19 vaccine, CD4 count, CD4/CD8 ratio, immune response

## Abstract

**Objectives**: Accumulating evidence has confirmed the efficacy and safety of COVID-19 vaccines against SARS-CoV-2 infection. However, the effect of COVID-19 vaccination on immuno-virological parameters in people with HIV (PWH) is uncertain. **Methods**: A total of 372 PWH treated at Beijing Ditan Hospital were included. Unvaccinated PWH were matched 1:3 with vaccinated PWH using a propensity score matching algorithm. Differences in immuno-virological markers between the matched groups were analyzed. The Wilcoxon signed rank test was used to test for changes in CD4 and CD8 counts and HIV viral load over two months around vaccination. In addition, we investigated the long-term changes in HIV-related markers in different vaccination dose groups and in the entire vaccinated population. **Results**: Vaccinated PWH had a higher CD4/CD8 ratio (0.64 (0.49, 0.78) vs. 0.80 (0.56, 1.03), *p* = 0.037) than unvaccinated PWH within a two-month window after the third dose. There were 337 PWH who received COVID-19 vaccination, and 73.9% (n = 249) received three doses of vaccine. We observed a transient increase in CD4 count and CD4/CD8 ratio within a two-month window after vaccination, especially after the second dose (CD4 count: 583.5 (428.5, 706.8) vs. 618.0 (452.0, 744.0), *p* = 0.018; CD4/CD8 ratio: 0.70 (0.50, 0.91) vs. 0.71 (0.53, 0.96), *p* < 0.001)) and the third dose (CD4 count: 575.5 (435.5, 717.0) vs. 577.5 (440.8, 754.8), *p* = 0.001; CD4/CD8 ratio: 0.70 (0.52, 0.93) vs. 0.79 (0.53, 1.00), *p* < 0.001)). Recent CD4 counts and CD4/CD8 ratios were lower than after COVID-19 but remained higher than before COVID-19 in vaccinated PWH. In addition, COVID-19 vaccination had no negative effect on HIV viral load. **Conclusions**: A transient increase in CD4 count and CD4/CD8 ratio was observed after COVID-19 vaccination. However, the enhanced cellular immune response induced by vaccination may diminish over time and return to normal levels. There is no adverse effect of vaccination on HIV viral load.

## 1. Introduction

Severe acute respiratory syndrome coronavirus 2 (SARS-CoV-2), a novel respiratory pathogen causing coronavirus disease 2019 (COVID-19), was first reported in Wuhan, China, in December 2019 [1]. Within months, COVID-19 had become a global pandemic, causing significant morbidity, mortality, and socio-economic disruption worldwide [2]. People with HIV (PWH) are recognized to be at high risk of SARS-CoV-2 infection [3]. Varying degrees of immunodeficiency and chronic inflammation may increase the incidence of serious health complications from COVID-19 compared with people without HIV [4].

Rapid development and administration of COVID-19 vaccine has significantly changed the trajectory of SARS-CoV-2 infection [5]. Accumulating evidence has confirmed that COVID-19 vaccination can elicit a satisfactory immune response in PWH comparable to that of the general population, except for cases with low CD4 count recovery [6]. Vaccination plays a critical protective role in reducing SARS-CoV-2 transmission, severity of infection, and COVID-19-related mortality in PWH [7,8]. Although the safety and immunogenicity of SARS-CoV-2 vaccine in PWH have been confirmed, there remain some concerns about its potential effects on HIV immuno-virological markers (CD4 and CD8 counts and HIV viral load) [9,10,11]. Nevertheless, a recent study demonstrated that COVID-19 vaccination can rapidly prime CD4 T spike-specific cells independently of antiviral therapy (ART), leading to a transient increase in CD4 count and a small drop in HIV viral load [12]. It is worth noting that the researchers did not include unvaccinated PWH as a control group to compare HIV-related marker changes during the COVID-19 pandemic.

Reports on the association between COVID-19 vaccine and HIV-related markers are limited. The primary objective of this study was to assess the impact of COVID-19 vaccination on CD4 and CD8 cell counts, HIV viral load, and the durability of cellular immune response. The secondary objective was to investigate whether there were differences in immuno-virological parameters between unvaccinated and vaccinated PWH.

## 2. Materials and Methods

### 2.1. Patient Population and Study Design

**Figure 1:**
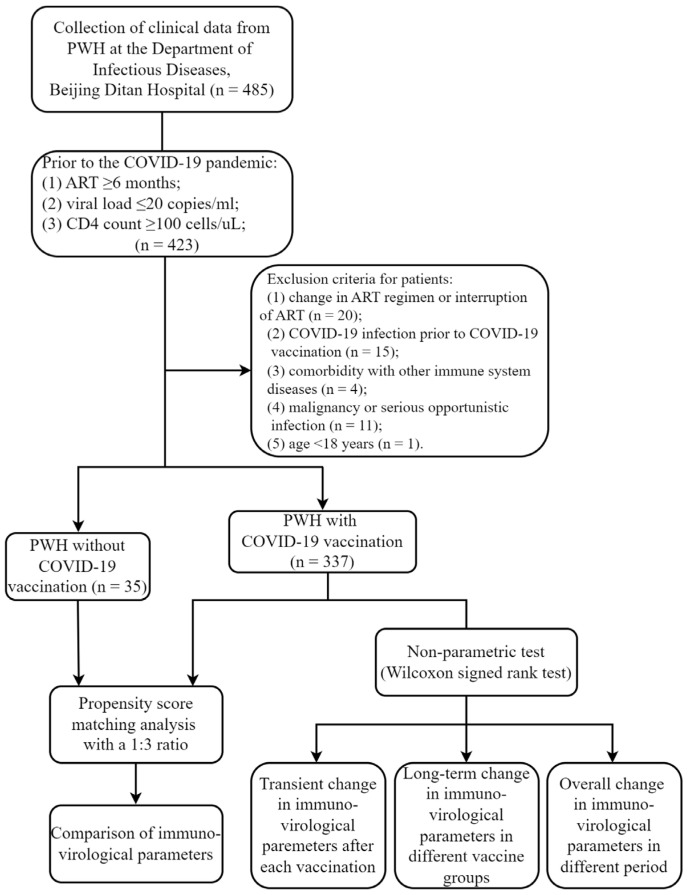
Flow chart for this study. PWH, people with HIV; COVID-19, coronavirus disease 2019; ART, antiretroviral therapy.

This retrospective cohort study was conducted in Beijing Ditan Hospital. PWH treated at the outpatient clinic of the Department of Infectious Diseases between January 2024 and June 2024 were enrolled. Prior to the COVID-19 pandemic, all PWH were on ART for ≥6 months and had undetectable HIV viral loads (≤20 copies/mL) and CD4 counts ≥ 100 cells/uL. Exclusion criteria were as follows: (1) change in ART regimen or interruption of ART (n = 20); (2) COVID-19 infection prior to COVID-19 vaccination (n = 15); (3) comorbidity with other immune system diseases (n = 4); (4) malignancy or serious opportunistic infection (n = 11); (5) age < 18 years (n = 1). Ultimately, 372 HIV-positive patients were enrolled in the present study (Figure 1).

### 2.2. Ethical Considerations

This study was approved by the institutional review board of Beijing Ditan Hospital (approval number: 2021-021-02) and performed in accordance with the ethical standards laid down in the 1964 Declaration of Helsinki and its later amendments. All patients provided written informed consent to participate in this study.

### 2.3. Data Collection

Demographic variables included age, sex, body mass index, personal history, and chronic complications. Laboratory tests included liver biochemical indicators (such as alanine aminotransferase and aspartate aminotransferase), renal function parameters (estimated glomerular filtration rate (eGFR) and creatinine), routine blood tests (such as hemoglobin and platelets), metabolic parameters (such as total cholesterol and glucose), and inflammatory indicators (C-reactive protein). 

In terms of immuno-virological markers, levels of CD4 count, CD8 count, and HIV viral load were collected from PWH at baseline, pre-COVID-19, approximately two months before and after each vaccination, post-COVID-19, and recently. In addition, we investigated information on the timing and dose of the COVID-19 vaccine received by patients.

According to the policy implemented in China, PWH are entitled to one free laboratory test every six months. In other words, changes in viral-immunological parameters are generally assessed every six months for PWH with stable conditions. Accordingly, the time frame before and after COVID-19 is based on a six-month period. Pre-COVID-19 refers to the period before the outbreak of COVID-19 (i.e., July to December 2019). Post-COVID-19 refers to the period after the end of the COVID-19 pandemic (i.e., January to June 2023). Recent refers to the period from January to June 2024.

### 2.4. Statistical Analysis

#### 2.4.1. Propensity Score Matching Analysis

As previously reported, propensity score matching is a valuable tool to reduce inherent selection bias and control for potential confounders in observational studies [13]. Therefore, the propensity score was calculated with an a priori logistic regression model based on 11 covariates, including age, sex, body mass index, smoking, drinking, chronic complications, and CD4 and CD8 counts before COVID-19. These covariates are listed in Table 1. Unvaccinated PWH were matched 1:3 with vaccinated patients. After propensity score matching, 130 observations (34 in the unvaccinated group and 96 in the vaccinated group) were retained for further analysis.

#### 2.4.2. Comparison of Variables

Continuous variables were expressed as mean ± standard deviation or median (interquartile range) in case of skewed distribution. Differences between groups were analyzed by Student’s *t* test or Mann–Whitney U test. Categorical variables were presented as percentages (%) and their statistical analysis was performed by the Chi-square test or Fisher’s exact test. A non-parametric test (Wilcoxon signed rank test) was used to compare the change in HIV viro-immunological markers before and after vaccination. Data analysis was performed with SPSS version 26.0. Figures were generated using R version 4.1.2. Statistical significance was set at *p* < 0.05.

### 2.5. Vaccines

The COVID-19 vaccines received by PWH were the inactivated vaccines (Vero cells) CoronaVac (Sinovac, Beijing, China) and Covilo (Sinopharm, Beijing, China). CoronaVac contains 3 µg in 0.5 mL of beta-propiolactone-inactivated SARS-CoV-2 from the CN02 strain grown in Vero cells and aluminum hydroxide as an adjuvant. Covilo contains 4 µg in 0.5 mL of beta-propiolactone-inactivated SARS-CoV-2 from the SARS-CoV-2019nCoV-CDC-Tan-HB02 strain grown in Vero cells and aluminum hydroxide as an adjuvant. Both inactivated vaccines were administered in doses of 0.5 ml [14,15]. Specifically, 180 PWH received CoronaVac vaccine and 157 received Covilo vaccine. 

## 3. Results

### 3.1. Baseline Characteristics

Baseline characteristics of PWH before and after propensity score matching are shown in Table 1. A total of 34 unvaccinated PWH were matched with 96 vaccinated PWH. In the two matched groups, the mean age was approximately 33 years, and most were male. The proportion of patients with chronic complications was low. In addition, the pre-COVID-19 CD4 count and CD4/CD8 ratio were similar between the two groups (*p* > 0.05). 

Supplementary Table S1 shows the laboratory characteristics in the unvaccinated and vaccinated PWH groups. There were no differences in liver function, kidney function, metabolic parameters, routine blood counts, and inflammatory indicators between the two groups (*p* > 0.05).

**Table 1:** Baseline characteristics.

Characteristics	Unadjusted			After Propensity Score Matching		
	Unvaccinated (n = 35)	Vaccinated (n = 337)	*p*	Unvaccinated (n = 34)	Vaccinated (n = 96)	*p*
Personal History						
Age (years)	33.4 ± 10.0	31.3 ± 8.4	0.160	33.5 ± 10.1	33.8 ± 8.8	0.866
Male, n (%)	34 (97.1)	325 (96.4)	0.767	33 (97.1)	93 (96.9)	1.000
BMI (kg/m^2^)	23.6 ± 2.0	23.7 ± 2.3	0.750	23.6 ± 2.0	23.5 ± 2.4	0.763
Smoking, n (%)	10 (28.6)	142 (42.1)	0.120	10 (29.4)	39 (40.6)	0.246
Drinking, n (%)	6 (17.1)	119 (35.3)	0.030	6 (17.6)	22 (22.9)	0.521
Comorbidities, n (%)						
Hypertension	1 (2.9)	14 (4.2)	0.710	1 (2.9)	1 (1.0)	0.456
Diabetes mellitus	5 (14.3)	25 (7.4)	0.183	4 (11.8)	10 (10.4)	0.759
Cardiovascular disease	1 (2.9)	5 (1.5)	0.450	1 (2.9)	1 (1.0)	0.456
Chronic kidney disease	5 (14.3)	32 (9.5)	0.371	5 (14.7)	13 (13.5)	1.000
Pre-COVID-19 HIV markers						
CD4 count	468.0 (356.0, 636.5)	562.0 (402.0, 740.0)	0.039	472.0 (389.0, 644.0)	490.0 (345.0, 659.0)	0.962
CD4/CD8 ratio	0.56 (0.44, 0.81)	0.65 (0.47, 0.89)	0.236	0.59 (0.44, 0.81)	0.58 (0.38, 0.79)	0.571

Values are mean ± standard deviation, number (percentage), or median (interquartile range). Abbreviations: BMI, body mass index; COVID-19, coronavirus disease 2019.

### 3.2. ART and HIV Markers in the Matched Cohort

Table 2 shows that the ART regimen was similar between the matched groups (*p* > 0.05). Unvaccinated PWH had similar primary infection rates (73.5% vs. 79.2%) and repeat infection rates (5.9% vs. 9.4%) compared to vaccinated individuals. Two months after the first vaccination, there was no significant difference in CD4 count and CD4/CD8 ratio between the two groups (*p* > 0.05). However, vaccinated PWH had a higher CD4/CD8 ratio (0.64 (0.49, 0.78) vs. 0.80 (0.56, 1.03), *p* = 0.037) than unvaccinated PWH within a two-month window after the third dose. Notably, only 13 unvaccinated PWH had laboratory tests in the two months after the vaccinated group received the second dose, so we did not analyze this time point. Although there was no statistical difference, post-COVID-19 and recent CD4 counts and CD4/CD8 ratios were slightly higher in vaccinated PWH. In addition, we found that there was no significant difference in the ratio of immunological parameters between post-COVID-19 and pre-COVID-19 (*p* > 0.05).

### 3.3. Changes in CD4 and CD8 Counts Before and After Vaccination

**Figure 2:**
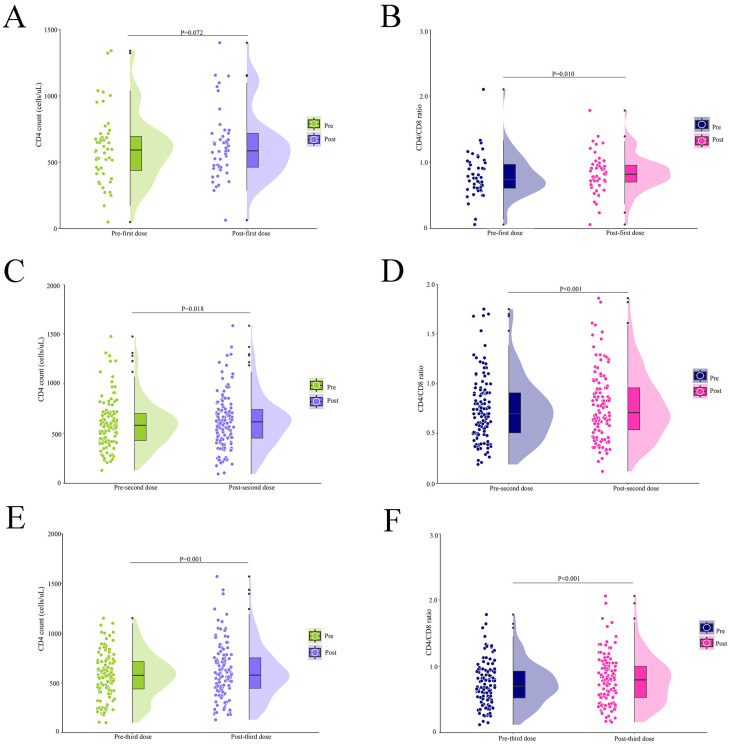
Median changes in CD4 count and CD4/CD8 ratio before and after vaccination: (**A**) CD4 count at first dose (n = 48); (**B**) CD4/CD8 ratio at first dose (n = 48); (**C**) CD4 count at second dose (n = 124); (**D**) CD4/CD8 ratio at second dose (n = 124); (**E**) CD4 count at third dose (n = 122); (**F**) CD4/CD8 ratio at third dose (n = 122).

Due to the impact of COVID-19 epidemic control, patients were unable to attend outpatient clinics for regular HIV immuno-virological marker testing. Only 48 PWH accepted testing within two months of the first vaccination. Although there was no significant change in CD4 count (592.5 (421.0, 709.0) vs. 586.0 (461.5, 718.0), *p* = 0.072), the CD4/CD8 ratio (0.74 (0.60, 0.98) vs. 0.82 (0.68, 0.96), *p* = 0.010) was significantly increased after vaccination (Figure 2A,B). Of those who received a second dose, 124 individuals underwent laboratory testing. There were significant differences in CD4 count (583.5 (428.5, 706.8) vs. 618.0 (452.0, 744.0), *p* = 0.018) and CD4/CD8 ratio (0.70 (0.50, 0.91) vs. 0.71 (0.53, 0.96), *p* < 0.001) (Figure 2C,D). After the third vaccination, 122 individuals underwent immuno-virological marker testing. CD4 cell count (575.5 (435.5, 717.0) vs. 577.5 (440.8, 754.8), *p* = 0.001) and CD4/CD8 ratio (0.70 (0.52, 0.93) vs. 0.79 (0.53, 1.00), *p* < 0.001) were higher than before vaccination (Figure 2E,F). 

**Figure 3:**
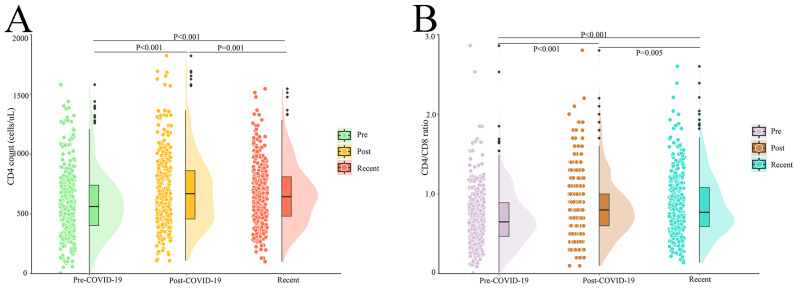
The levels of CD4 count and CD4/CD8 ratio at pre-COVID-19, post-COVID-19, and recently in PWH receiving vaccination (n = 337): (**A**) CD4 count at different time points; (**B**) CD4/CD8 ratio at different time points. COVID-19, coronavirus disease 2019; PWH, people with HIV.

We compared the difference in immunological parameters between two months after each vaccination and the post-COVID-19 period. However, there was no significant difference in CD4 count and CD4/CD8 ratio between post-vaccination and post-COVID-19 (*p* > 0.05) (Supplementary Table S2). Regarding the overall change in HIV markers in 337 vaccinated PWH, we found that the most recent CD4 count (644.0 (478.5, 813.0) vs. 669.0 (456.0, 861.5), *p* = 0.001) and CD4/CD8 ratio (0.77 (0.59, 1.08) vs. 0.79 (0.59, 0. 99), *p* = 0.005) were lower than after COVID-19 but remained higher than the pre-COVID-19 CD4 count (644.0 (478.5, 813.0) vs. 562.0 (401.5, 741.0), *p* < 0.001) and CD4/CD8 ratio (0.77 (0.59, 1.08) vs. 0.65 (0.47, 0.90), *p* < 0.001) (Figure 3A,B). Notably, there was no significant difference in immuno-virological parameters between the two inactivated vaccines.

Similar results were observed in the subgroup analysis, as described in Table 3. Higher levels of CD4 count and CD4/CD8 ratio were observed after vaccination in the group with CD4 count > 500 cells/uL, CD4/CD8 ratio > 1, ART initiation > 1 year, and no SARS-CoV-2 infection.

### 3.4. Association Between Changes in CD4 Count and CD4/CD8 Ratio and Vaccine Doses

A total of 337 PWH received COVID-19 vaccine, of which 11 received one dose of vaccine, 77 received two doses, and 249 received three doses. There were no differences in personal history and comorbidities between PWH with different vaccination doses. CD4 count, CD8 count, and CD4/CD8 ratio were similar between the dose groups at baseline and before COVID-19 (*p* > 0.05). The median time of ART initiation was four years among vaccinated PWH (Supplementary Table S3).

**Figure 4:**
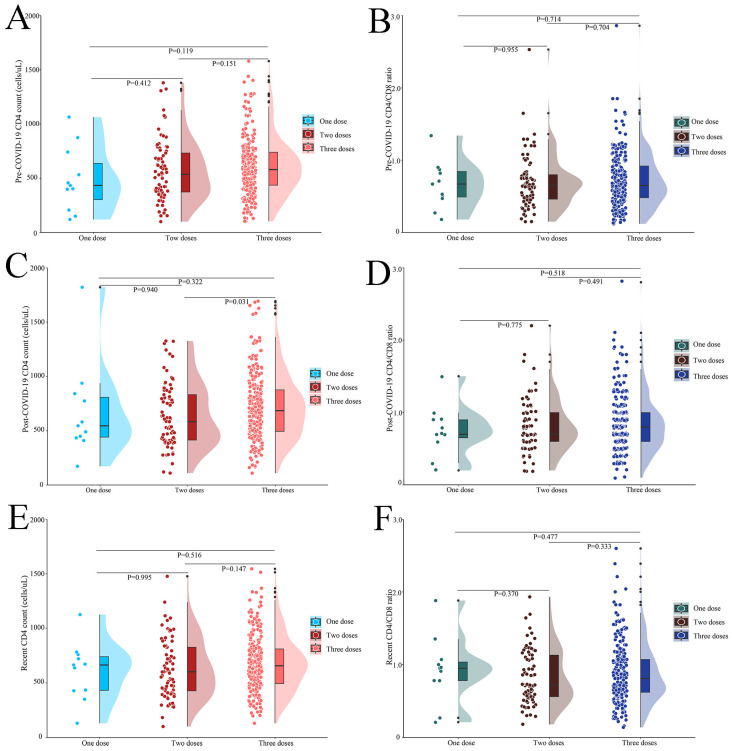
CD4 count and CD4/CD8 ratio at different vaccination doses and time points (n = 337): (**A**) pre-COVID-19 CD4 count at different vaccination doses; (**B**) pre-COVID-19 CD4/CD8 ratio at different vaccination doses; (**C**) post-COVID-19 CD4 count at different vaccination doses; (**D**) post-COVID-19 CD4/CD8 ratio at different vaccination doses; (**E**) recent CD4 count at different vaccination doses; (**F**) recent CD4/CD8 ratio at different vaccination doses. COVID-19, coronavirus disease 2019.

As shown in Supplementary Table S4 and Figure 4, the three groups had similar CD4 counts and CD4/CD8 ratios before COVID-19 (all *p* > 0.05) (Figure 4A,B). PWH who received three doses of vaccine had higher post-pandemic CD4 counts than those who received two doses (685.0 (494.0, 877.0) vs. 583.0 (414.0, 833.0), *p* = 0.031) (Figure 4C). However, there was no significant difference in the CD4/CD8 ratio between the three groups (*p* > 0.05) (Figure 4D). According to recent laboratory tests, the three groups had similar CD4 counts and CD4/CD8 ratios (all *p* > 0.05) (Figure 4E,F).

### 3.5. HIV Viral Load During the Epidemic

In this study, we found no adverse effect of COVID-19 vaccination or SARS-CoV-2 infection on HIV viral load. As we only included PWH with viral control ≤20 copies/mL, data on undetectable HIV viral load are not shown in figures or tables throughout this study.

### 3.6. Changes in Biochemical Indicators in PWH with Previous COVID-19 Infection

We evaluated the long-term effects of COVID-19 infection on liver and kidney function, metabolism, and inflammation in PWH. There were no significant differences in serum levels of alanine aminotransferase, aspartate aminotransferase, glucose, total cholesterol, triglyceride, and C-reactive protein before and after COVID-19. Albumin and eGFR levels were lower after COVID-19 than before COVID-19, but the magnitude of the change may be of negligible clinical significance (Supplementary Table S5).

## 4. Discussion

In this study, we found that the following: (1) approximately 90.6% (337 cases) of PWH received COVID-19 vaccination, of whom 11 received one dose, 77 received two doses, and 249 received three doses; (2) there was a transient increase in CD4 counts and CD4/CD8 ratios within two months of vaccination, especially after the second and third doses; (3) PWH who received three vaccine doses had higher post-pandemic CD4 counts than those who received two doses and one dose; (4) although recent CD4 counts and CD4/CD8 ratios were lower than after COVID-19, they were still higher than before the pandemic; (5) vaccinated PWH had a higher CD4/CD8 ratio than unvaccinated PWH within a two-month window after the third dose, but the two groups had similar CD4 counts and CD4/CD8 ratios in the post-pandemic and recent periods. 

Vaccination is recognized as one of the most effective public health interventions against the COVID-19 pandemic [16,17]. COVID-19 vaccine is recommended for PWH, especially those with CD4 count <200 cells/uL, unsuppressed/detectable HIV viral load, or opportunistic infection, who are considered a high-priority group for vaccination [18]. It is well established that COVID-19 vaccine played a key role in protecting PWH against SARS-CoV-2 infection, reducing virus transmission, infection severity, and COVID-19-associated mortality [19,20]. However, limited evidence suggests that vaccines may have adverse effects on HIV immuno-virological markers. Previous reports have shown that streptococcus pneumonia, hepatitis B virus, and influenza vaccinations can cause transient increases in HIV viral load [21,22,23]. In addition, COVID-19 vaccine may induce suboptimal immune responses in PWH due to persistent immune dysfunction and exhaustion, even after HIV suppression and CD4 cell restoration with ART [24]. Therefore, it is necessary to evaluate the potential impact of COVID-19 vaccination on HIV immunity and virology.

To the best of our knowledge, this study is the first to analyze the association between immuno-virological markers and SARS-CoV-2 vaccination in Chinese PWH during the three-year COVID-19 pandemic. We found that 337 (90.6%) PWH received COVID-19 vaccination, of which 11 received one dose, 77 received two doses, and 249 received three doses. A meta-analysis of COVID-19 vaccine uptake showed that approximately 50% of PWH worldwide received at least one dose of the vaccine; stratified by WHO region, uptake was highest in the European region at 90.1%, followed by the Southeast Asian region at 78.9% and the Americas at 71.6% [25]. COVID-19 vaccine hesitancy and uptake among PWH remains highly variable in different WHO regions and socio-demographic contexts around the world. Evidence suggests that the rate of COVID-19 vaccine hesitancy in PWH is significantly higher than in HIV-uninfected individuals [26,27]. One of the most important concerns may be the potential effects of the vaccine on HIV immuno-virological parameters.

We observed a transient increase in CD4 count and CD4/CD8 ratio over a two-month window following COVID-19 vaccination, particularly after the second and third doses. Our previous studies have shown that COVID-19 booster shots are effective in PWH, resulting in increases in CD4 cell counts and neutralizing antibodies that last for up to six months [28]. Consistent with our findings, a recent study from Italy also showed a transient increase in CD4 count after COVID-19 vaccination, and the results remained after restricting the analysis to virosuppressed PWH with CD4 ≤200 cells/uL and more than six months of ART prior to vaccination [12]. In addition, vaccine-induced spike-specific T-cell responses are equivalent after two or three doses of COVID-19 vaccination in PWH and HIV-negative controls [29]. This suggests that the transient increase in CD4 cell frequencies may be associated with rapid priming of CD4 T spike-specific cells by COVID-19 vaccine, independent of ART [30,31]. The observed association between CD4 cell count and COVID-19 vaccine is not an epiphenomenon; rather, vaccination may have a direct potentiating effect on cellular immune responses in PWH.

In the subgroup analysis, we found that CD4 cell counts and CD4/CD8 ratios were also increased after vaccination in PWH on ART >1 year or with CD4 cell counts >500 cells/uL. In terms of overall HIV marker fluctuation, recent CD4 counts and CD4/CD8 ratios were lower than in the post-pandemic period but remained higher than before COVID-19 in the vaccinated PWH group. In addition, we compared changes in HIV immunological parameters between vaccinated and unvaccinated PWH using propensity score matching analysis. Vaccinated PWH had a higher CD4/CD8 ratio than unvaccinated PWH within a two-month window after the third dose. Although vaccinated PWH had slightly elevated CD4 counts and CD4/CD8 ratios compared with unvaccinated controls in the post-pandemic and recent periods, the magnitude of the changes was not statistically different. This phenomenon may be related to the following reasons. First, we included a small sample size of 34 unvaccinated PWH, which may limit the statistical power of this study. Second, the difference in immunological parameters between the two groups diminished as the level of vaccination-induced cellular response decreased over time. A collection of evidence from available studies also suggests that T-cell responses are well maintained six months after COVID-19 vaccination, whereas humoral responses appear to be significantly attenuated six months after initial vaccination [32,33,34]. It is now well established that the humoral and cellular responses elicited by either natural SAR-CoV-2 infection or vaccination in PWH wane significantly over time [35]. However, the durability and waning rate of immune response varies between different vaccine regimens, and booster doses may compensate for waning efficacy [36,37]. 

An additional dose of SARS-CoV-2 vaccine not only boosts immunity but also prolongs protection [38]. Global guidelines also recommend extending the COVID-19 vaccine series in PWH and providing booster doses in moderate to severe immunosuppression [18,39]. We observed an increasing trend in CD4 count and CD4/CD8 ratio with extended vaccine doses, with PWH in the three-dose vaccine group having higher post-COVID-19 CD4 counts than those in the two-dose and one-dose groups. Previous studies have shown that a third dose of SARS-CoV-2 mRNA vaccine significantly enhances the immune response in PWH, including those with advanced HIV infection [40,41]. Repeated exposure to SARS-CoV-2 antigen appears to have a maturational effect, inducing both enhanced T-cell effector function and diverse response potential to spike proteins and other SARS-CoV-2 internal proteins [42]. In addition, clinical evidence suggests that a third dose of vaccine effectively reduces the risk of breakthrough infection in PWH [43]. Thus, repeated exposure to SARS-CoV-2 (either by infection or vaccination) may enhance immune responses in PWH [44]. 

Not only the immune response but also the HIV viral load may be affected by COVID-19 vaccination. Reports by Vergori et al. [12] suggest a small decrease in HIV viral load within one month of COVID-19 vaccination in PWH. However, Matveev et al. [45] found a slight increase in the frequency of detectable viremia after the two doses of COVID-19 vaccine in older PWH. Even without HIV infection, advanced age is associated with an increased risk of immune dysfunction and immunosenescence [46,47]. Thus, HIV viral load may take the opportunity to increase when the compromised immune system struggles to mobilize in response to the SARS-CoV-2 vaccine in the elderly HIV population. In contrast to the findings of the two previous studies, our study found no apparent change in virological parameters in PWH during the COVID-19 pandemic. Because only PWH with an undetectable HIV viral load (≤20 copies/ml) were included in this study, we were unable to assess whether vaccine responses could lead to further reductions in HIV viral load. It is certain that neither vaccination nor SARS-CoV-2 infection caused virological failure in the present study.

The SARS-CoV-2 virus attacks not only the lungs but also other organs such as the liver and kidneys, leading to serious complications [48]. Previous reports have focused on organ damage during the acute phase of SARS-CoV-2 infection, but whether this damage is long-lasting in immunosuppressed populations is unclear [49,50]. Our study investigated the most recent levels of liver and kidney function parameters, metabolic markers, and inflammatory markers in PWH with previous COVID-19 infection. Although a statistically significant decrease in eGFR and albumin levels was observed, the magnitude of the changes was of negligible clinical significance. There were no significant effects of SARS-CoV-2 virus on other biomarkers. However, our study only included PWH with CD4 >100 cells/uL and viral suppression, so the results are not representative of the whole HIV population. In addition, the small sample size may lead to some random effects and false-negative results. Given the potential for chronic and systemic damage left by SARS-CoV-2 infection, future studies with long-term follow-up need to investigate the possible effects of long COVID in PWH [51]. 

Vaccination protects against the severe SARS-CoV-2 infection in PWH, but its effect on the state of general immunity, including CD4 cells, CD4/CD8 ratio, and HIV viral load, is not fully understood. Although COVID-19 has become part of a long list of common infectious respiratory diseases, some PWH remain at risk of re-infection and severe infection [52]. In fact, a significant proportion of PWH have COVID-19 vaccine hesitancy and remain unvaccinated [53]. Therefore, it is necessary to investigate the long-term effects of COVID-19 vaccine on PWH. In this study, we first evaluated the changes in immuno-virological parameters at a median of three years after COVID-19 vaccination in Chinese PWH. We found that the SARS-CoV-2 vaccine induced a good immune response in PWH: a transient increase in CD4 count and CD4/CD8 ratio over a two-month window after COVID-19 vaccination. In addition, there were no adverse effects on immuno-virological parameters over a median follow-up of three years. Our results may provide evidence for the long-term safety of COVID-19 vaccine, especially with regard to immuno-virological parameters.

Some limitations should be noted. First, we included a small sample size of PWH, which makes our results less conclusive and comprehensive. Second, HIV-related markers were assessed approximately two months after vaccination; such a long interval may mask the true change in these markers. Third, due to the impact of epidemic containment, only a subset of patients received regular viro-immunological marker testing after each vaccine dose, further limiting the accuracy and reliability of the results. In addition, some of the PWH with advanced immunosuppression (pre-COVID-19 CD4 count 100–350 cells/uL) were included, so the increases in CD4 counts may be partly related to ART. Finally, we did not evaluate the association between vaccination and clinical efficacy endpoints, such as SARS-CoV-2 infection rates and severity of infection.

## 5. Conclusions

This study presented real-world data showing that there was no adverse effect of COVID-19 vaccine on immuno-virological markers in PWH. In contrast, we found a transient increase in CD4 count and CD4/CD8 ratio after COVID-19 vaccination. However, the cellular immune response induced by vaccination may attenuate over time and return to normal levels. Our results provide long-term evidence of the safety of COVID-19 vaccination, particularly in terms of immuno-virological parameters, which is a positive message for future campaigns. Future studies are needed to investigate the impact of COVID-19 vaccination on the immune response in PWH, including vaccine modalities, doses required, and time interval.

## Figures and Tables

**Table 2:** Comparison of ART regimen and HIV-related markers between the matched groups.

Characteristics	Unvaccinated (n = 34)	Vaccinated (n = 96)	*p*
ART, n (%)			
TDF/AZT+3TC+EFV/NVP	28 (82.4)	75 (78.1)	0.601
TDF/AZT+3TC+LPV/r	4 (11.8)	10 (10.4)	0.759
Compound agents (BIC/FTC/TAF)	1 (2.9)	5 (5.2)	1.000
Compound agents (DTG/3TC)	1 (2.9)	6 (6.3)	0.675
Frequency of COVID-19 infection, n (%)			
0	7 (20.6)	11 (11.4)	0.372
1	25 (73.5)	76 (79.2)	
2	2 (5.9)	9 (9.4)	
HIV markers			
Two months after first dose			
CD4 count, cells/uL	500.0 (401.0, 676.0)	579.0 (455.5, 709.0)	0.511
CD8 count, cells/uL	808.5 (680.0, 958.0)	780.0 (579.5, 1002.0)	0.585
CD4/CD8 ratio	0.65 (0.57, 0.78)	0.80 (0.68, 0.95)	0.315
Two months after third dose			
CD4 count, cells/uL	540.0 (443.5, 694.0)	569,9 (442.0, 749.5)	0.554
CD8 count, cells/uL	903.5 (690.0, 1000.0)	807.5 (628.0, 981.0)	0.185
CD4/CD8 ratio	0.64 (0.49, 0.78)	0.80 (0.56, 1.03)	0.037
Post-COVID-19			
CD4 count, cells/uL	613.0 (465.0, 757.0)	630.0 (444.0, 811.5)	0.803
CD8 count, cells/uL	769.0 (606.0, 1099.0)	811.5 (573.0, 1038.0)	0.947
CD4/CD8 ratio	0.70 (0.56, 0.92)	0.75 (0.53, 0.96)	0.677
Recent			
CD4 count, cells/uL	589.5 (408.0, 742.0)	622.5 (470.5, 808.0)	0.343
CD8 count, cells/uL	708.0 (576.0, 957.0)	762.0 (575.5, 1021.0)	0.470
CD4/CD8 ratio	0.73 (0.63, 0.97)	0.76 (0.61, 1.07)	0.843
Ratio of post-COVID-19 to pre-COVID-19			
Post-CD4/pre-CD4 ratio	1.12 (1.01, 1.55)	1.17 (1.05, 1.37)	0.651
(Post-CD4/CD8)/(pre-CD4/CD8) ratio	1.11 (1.01, 1.42)	1.20 (1.03, 1.42)	0.573
Ratio of recent to pre-COVID-19			
Recent-CD4/pre-CD4 ratio	1.10 (0.97, 1.53)	1.20 (0.98, 1.41)	0.637
(Recent-CD4/CD8)/(pre-CD4/CD8) ratio	1.27 (1.05, 1.67)	1.31 (1.08, 1.55)	0.739

Values are number (percentage) or median (interquartile range). Abbreviations: ART, antiretroviral therapy; COVID-19, coronavirus disease 2019.

**Table 3:** Changes in HIV markers in subgroups.

Characteristics	Pre-COVID-19	Post-Vaccination	*p*
CD4 count > 500 cells/uL (n = 207)			
CD4 count	695.0 (598.0, 820.0)	803.0 (669.0, 988.0)	<0.001
CD8 count	981.0 (760.0, 1245.0)	902.0 (700.0, 1174.0)	0.432
CD4/CD8 ratio	0.77 (0.59, 1.01)	0.89 (0.69, 1.13)	<0.001
CD4/CD8 ratio > 1 (n = 69)			
CD4 count	701.0 (561.0, 1044.5)	848.0 (670.5, 1124.0)	<0.001
CD8 count	632.0 (443.0, 821.0)	658.0 (528.5, 958.0)	0.009
CD4/CD8 ratio	1.18 (1.06, 1.29)	1.27 (1.09, 1.52)	0.007
ART initiation > 1 year (n = 263)			
CD4 count	589.0 (422.0, 765.0)	690.0 (488.0, 888.0)	<0.001
CD8 count	857.0 (639.0, 1131.0)	823.0 (631.0, 1097.0)	0.353
CD4/CD8 ratio	0.68 (0.48, 0.98)	0.81 (0.61, 1.05)	<0.001
No COVID-19 infection (n = 37)			
CD4 count	600.0 (409.0, 750.5)	783.0 (430.5, 878.0)	<0.001
CD8 count	861.0 (518.0, 1251.5)	769.0 (565.5, 1341.0)	0.592
CD4/CD8 ratio	0.73 (0.53, 1.00)	0.84 (0.63, 1.24)	<0.001

Values are median (interquartile range). Abbreviations: COVID-19, coronavirus disease 2019; ART, antiretroviral therapy.

## Data Availability

The datasets generated and analyzed during the present study are not publicly available due to the restrictions imposed by the Beijing Ditan Hospital who is the data owner. The authors used this dataset under an agreement with the Beijing Ditan Hospital for the current study. If anyone wants to access the data used in the study for reasonable requests, please contact the corresponding author, Xin Li.

## Supplementary Materials

The following supporting information can be downloaded at: https://www.mdpi.com/article/10.3390/vaccines12121365/s1, Table S1: Pre-COVID-19 biochemical indicators between the matched groups; Table S2: Comparison of CD4/CD8 counts between post-vaccination and post-COVID-19; Table S3: Baseline information on PWH receiving different vaccination doses; Table S4: HIV markers in PWH receiving different vaccination doses; Table S5: Changes in biochemical indicators in PWH with previous COVID-19 infection.

## References

[1] Markov P.V., Ghafari M., Beer M., Lythgoe K., Simmonds P., Stilianakis N.I., Katzourakis A. (2023). The evolution of SARS-CoV-2. Nat. Rev. Microbiol..

[2] Bash K., Sacha G., Latifi M. (2023). COVID-19: A management update. Cleve. Clin. J. Med..

[3] Brolly J., Chadwick D.R. (2023). COVID-19 infection in people living with HIV. Br. Med. Bull..

[4] Thornhill J., Orkin C., Cevik M. (2023). Estimating the global impact of coronavirus disease 2019 on people living with HIV. Curr. Opin. Infect. Dis..

[5] Ao D., He X., Liu J., Xu L. (2023). Strategies for the development and approval of COVID-19 vaccines and therapeutics in the post-pandemic period. Signal Transduct. Target. Ther..

[6] Levy I., Rahav G. (2023). The effect of HIV on COVID-19 vaccine responses. Curr. Opin. HIV AIDS.

[7] Salo J., Hägg M., Kortelainen M., Leino T., Saxell T., Siikanen M., Sääksvuori L. (2022). The indirect effect of mRNA-based COVID-19 vaccination on healthcare workers’ unvaccinated household members. Nat. Commun..

[8] Kelly J.D., Leonard S., Hoggatt K.J., Boscardin W.J., Lum E.N., Moss-Vazquez T.A., Andino R., Wong J.K., Byers A., Bravata D.M. (2022). Incidence of Severe COVID-19 Illness Following Vaccination and Booster With BNT162b2, mRNA-1273, and Ad26.COV2.S Vaccines. JAMA.

[9] Bozzi G., Lombardi A., Ludovisi S., Muscatello A., Manganaro L., Cattaneo D., Gori A., Bandera A. (2021). Transient increase in plasma HIV RNA after COVID-19 vaccination with mRNA-1272. Int. J. Infect. Dis..

[10] Levy I., Wieder-Finesod A., Litchevsky V., Biber A., Indenbaum V., Olmer L., Huppert A., Mor O., Goldstein M., Levin E.G. (2021). Immunogenicity and safety of the BNT162b2 mRNA COVID-19 vaccine in people living with HIV-1. Clin. Microbiol. Infect..

[11] Fusco F.M., Carleo M.A., Sangiovanni N., D’Abbraccio M., Tambaro O., Borrelli F., Viglietti R., Camaioni C., Bruner V., Falanga R. (2023). Does COVID-19 Vaccination with BNT162b2 Influence HIV-Related Immunological and Virological Markers? Data from 235 Persons Living with HIV at Cotugno Hospital, Naples, Italy: Immune Response After Second and Third Doses, and Influence on Immunovirological Markers. Viral Immunol..

[12] Vergori A., Cozzi-Lepri A., Tavelli A., Mazzotta V., Azzini A.M., Gagliardini R., Mastrorosa I., Latini A., Pellicanò G., Taramasso L. (2024). SARS-CoV-2 mRNA vaccination and short-term changes in viral load and CD4/CD8 T-cell counts in people living with HIV. Int. J. Infect. Dis..

[13] Chen J.W., Maldonado D.R., Kowalski B.L., Miecznikowski K.B., Kyin C., Gornbein J.A., Domb B.G. (2022). Best Practice Guidelines for Propensity Score Methods in Medical Research: Consideration on Theory, Implementation, and Reporting. A Review. Arthroscopy.

[14] Al Kaabi N., Zhang Y., Xia S., Yang Y., Al Qahtani M.M., Abdulrazzaq N., Al Nusair M., Hassany M., Jawad J.S., Abdalla J. (2021). Effect of 2 Inactivated SARS-CoV-2 Vaccines on Symptomatic COVID-19 Infection in Adults: A Randomized Clinical Trial. JAMA.

[15] Hua Q., Zhang H., Yao P., Xu N., Sun Y., Lu H., Xu F., Liao Y., Yang J., Mao H. (2022). Immunogenicity and immune-persistence of the CoronaVac or Covilo inactivated COVID-19 Vaccine: A 6-month population-based cohort study. Front. Immunol..

[16] Zhang Y., Zeng G., Pan H., Li C., Hu Y., Chu K., Han W., Chen Z., Tang R., Yin W. (2021). Safety, tolerability, and immunogenicity of an inactivated SARS-CoV-2 vaccine in healthy adults aged 18–59 years: A randomised, double-blind, placebo-controlled, phase 1/2 clinical trial. Lancet Infect. Dis..

[17] Zheng C., Shao W., Chen X., Zhang B., Wang G., Zhang W. (2022). Real-world effectiveness of COVID-19 vaccines: A literature review and meta-analysis. Int. J. Infect. Dis..

[18] CDC (2023). COVID-19 Vaccines for People Who Are Moderately or Severely Immunocompromised. https://www.cdc.gov/coronavirus/2019-ncov/vaccines/recommendations/immuno.html.

[19] Beladiya J., Kumar A., Vasava Y., Parmar K., Patel D., Patel S., Dholakia S., Sheth D., Boddu S.H.S., Patel C. (2024). Safety and efficacy of COVID-19 vaccines: A systematic review and meta-analysis of controlled and randomized clinical trials. Rev. Med. Virol..

[20] Griffin D.W.J., Pai Mangalore R., Hoy J.F., McMahon J.H. (2023). Immunogenicity, effectiveness, and safety of SARS-CoV-2 vaccination in people with HIV. AIDS..

[21] Negredo E., Domingo P., Sambeat M.A., Rabella N., Vázquez G. (2001). Effect of pneumococcal vaccine on plasma HIV-1 RNA of stable patients undergoing effective highly active antiretroviral therapy. Eur. J. Clin. Microbiol. Infect. Dis..

[22] Rey D., Krantz V., Partisani M., Schmitt M.P., Meyer P., Libbrecht E., Wendling M.J., Vetter D., Nicolle M., Kempf-Durepaire G. (2000). Increasing the number of hepatitis B vaccine injections augments anti-HBs response rate in HIV-infected patients. Effects on HIV-1 viral load. Vaccine.

[23] Günthard H.F., Wong J.K., Spina C.A., Ignacio C., Kwok S., Christopherson C., Hwang J., Haubrich R., Havlir D., Richman D.D. (2000). Effect of influenza vaccination on viral replication and immune response in persons infected with human immunodeficiency virus receiving potent antiretroviral therapy. J. Infect. Dis..

[24] Höft M.A., Burgers W.A., Riou C. (2024). The immune response to SARS-CoV-2 in people with HIV. Cell Mol. Immunol..

[25] Sulaiman S.K., Musa M.S., Tsiga-Ahmed F.I., Sulaiman A.K., Bako A.T. (2024). A systematic review and meta-analysis of the global prevalence and determinants of COVID-19 vaccine acceptance and uptake in people living with HIV. Nat. Hum. Behav..

[26] Kaida A., Brotto L.A., Murray M.C.M., Côté H.C.F., Albert A.Y., Nicholson V., Gormley R., Gordon S., Booth A., Smithm L.W. (2022). Intention to Receive a COVID-19 Vaccine by HIV Status Among a Population-Based Sample of Women and Gender Diverse Individuals in British Columbia, Canada. AIDS Behav..

[27] Pereira M., Santos Aleluia I.R., de Castro C.T., de Almeida Oliveira T., Cunha M.S., Magno L., Dourado I., Barreto F., Natividade M., Appiah S.C.Y. (2024). COVID-19 Vaccine Acceptance and Hesitancy among People Living with HIV: Review and Meta-Analysis. AIDS Behav..

[28] Yi Y., Han X., Cui X., Wang P., Wang X., Liu H., Wang Y., Zhu N., Li Y., Lin Y. (2023). Safety and Immunogenicity of the Inactivated COVID-19 Vaccine Booster in People Living with HIV in China. Vaccines.

[29] Datwani S., Kalikawe R., Waterworth R., Mwimanzi F.M., Liang R., Sang Y., Lapointe H.R. (2024). T-Cell Responses to COVID-19 Vaccines and Breakthrough Infection in People Living with HIV Receiving Antiretroviral Therapy. Viruses.

[30] Frater J., Ewer K.J., Ogbe A., Pace M., Adele S., Adland E., Alagaratnam J., Aley P.K., Ali M., Ansari M.A. (2021). Safety and immunogenicity of the ChAdOx1 nCoV-19 (AZD1222) vaccine against SARS-CoV-2 in HIV infection: A single-arm substudy of a phase 2/3 clinical trial. Lancet HIV.

[31] Antinori A., Cicalini S., Meschi S., Bordoni V., Lorenzini P., Vergori A., Lanini S., De Pascale L., Matusali G., Mariotti D. (2022). Humoral and Cellular Immune Response Elicited by mRNA Vaccination Against Severe Acute Respiratory Syndrome Coronavirus 2 (SARS-CoV-2) in People Living with Human Immunodeficiency Virus Receiving Antiretroviral Therapy Based on Current CD4 T-Lymphocyte Count. Clin. Infect. Dis..

[32] Woldemeskel B.A., Karaba A.H., Garliss C.C., Beck E.J., Wang K.H., Laeyendecker O., Cox A.L., Blankson J.N. (2022). The BNT162b2 mRNA Vaccine Elicits Robust Humoral and Cellular Immune Responses in People Living with Human Immunodeficiency Virus (HIV). Clin. Infect. Dis..

[33] Tuan J.J., Zapata H., Critch-Gilfillan T., Ryall L., Turcotte B., Mutic S., Andrews L., Roh M.E., Friedland G., Barakat L. (2022). Qualitative assessment of anti-SARS-CoV-2 spike protein immunogenicity (QUASI) after COVID-19 vaccination in older people living with HIV. HIV Med..

[34] Ogbe A., Pace M., Bittaye M., Tipoe T., Adele S., Alagaratnam J., Aley P.K., Ansari M.A., Bara A., Broadhead S. (2022). Durability of ChAdOx1 nCoV-19 vaccination in people living with HIV. JCI Insight.

[35] Goel R.R., Painter M.M., Apostolidis S.A., Mathew D., Meng W., Rosenfeld A.M., Lundgreen K.A., Reynaldi A., Khoury D.S., Pattekar A. (2021). mRNA vaccines induce durable immune memory to SARS-CoV-2 and variants of concern. Science.

[36] Au W.Y., Cheung P.P. (2022). Effectiveness of heterologous and homologous COVID-19 vaccine regimens: Living systematic review with network meta-analysis. BMJ.

[37] Higdon M.M., Baidya A., Walter K.K., Patel M.K., Issa H., Espié E., Feikin D.R., Knoll M.D. (2022). Duration of effectiveness of vaccination against COVID-19 caused by the omicron variant. Lancet Infect. Dis..

[38] Chan D.P.C., Wong N.S., Wong B.C.K., Chan J.M.C., Lee S.S. (2022). Three-Dose Primary Series of Inactivated COVID-19 Vaccine for Persons Living with HIV, Hong Kong. Emerg. Infect. Dis..

[39] Yin J., Chen Y., Li Y., Wang C., Zhang X. (2022). Immunogenicity and efficacy of COVID-19 vaccines in people living with HIV: A systematic review and meta-analysis. Int. J. Infect. Dis..

[40] Lapointe H.R., Mwimanzi F., Cheung P.K., Sang Y., Yaseen F., Umviligihozo G., Kalikawe R., Speckmaier S., Moran-Garcia N., Datwani S. (2023). People With Human Immunodeficiency Virus Receiving Suppressive Antiretroviral Therapy Show Typical Antibody Durability After Dual Coronavirus Disease 2019 Vaccination and Strong Third Dose Responses. J. Infect. Dis..

[41] Vergori A., Cozzi Lepri A., Cicalini S., Matusali G., Bordoni V., Lanini S., Meschi S., Iannazzo R., Mazzotta V., Colavita F. (2022). Immunogenicity to COVID-19 mRNA vaccine third dose in people living with HIV. Nat. Commun..

[42] Minervina A.A., Pogorelyy M.V., Kirk A.M., Crawford J.C., Allen E.K., Chou C.H., Mettelman R.C., Allison K.J., Lin C.Y., Brice D.C. (2022). SARS-CoV-2 antigen exposure history shapes phenotypes and specificity of memory CD8+ T cells. Nat. Immunol..

[43] Coburn S.B., Humes E., Lang R., Stewart C., Hogan B.C., Gebo K.A., Napravnik S., Edwards J.K., Browne L.E., Park L.S. (2022). Analysis of Postvaccination Breakthrough COVID-19 Infections Among Adults with HIV in the United States. JAMA Netw. Open.

[44] Keeton R., Tincho M.B., Suzuki A., Benede N., Ngomti A., Baguma R., Chauke M.V., Mennen M., Skelem S., Adriaanse M. (2023). Impact of SARS-CoV-2 exposure history on the T cell and IgG response. Cell Rep. Med..

[45] Matveev V.A., Mihelic E.Z., Benko E., Budylowski P., Grocott S., Lee T., Korosec C.S., Colwill K., Stephenson H., Law R. (2023). Immunogenicity of COVID-19 vaccines and their effect on HIV reservoir in older people with HIV. iScience.

[46] Calcinotto A., Kohli J., Zagato E., Pellegrini L., Demaria M., Alimonti A. (2019). Cellular Senescence: Aging, Cancer, and Injury. Physiol. Rev..

[47] Brauning A., Rae M., Zhu G., Fulton E., Admasu T.D., Stolzing A., Sharma A. (2022). Aging of the Immune System: Focus on Natural Killer Cells Phenotype and Functions. Cells.

[48] Gupta A., Madhavan M.V., Sehgal K., Nair N., Mahajan S., Sehrawat T.S., Bikdeli B., Ahluwalia N., Ausiello J.C., Wan E.Y. (2020). Extrapulmonary manifestations of COVID-19. Nat. Med..

[49] Zhang J.J., Dong X., Liu G.H., Gao Y.D. (2023). Risk and Protective Factors for COVID-19 Morbidity, Severity, and Mortality. Clin. Rev. Allergy Immunol..

[50] Hu W.S., Jiang F.Y., Shu W., Zhao R., Cao J.M., Wang D.P. (2022). Liver injury in COVID-19: A minireview. World J. Gastroenterol..

[51] Ely E.W., Brown L.M., Fineberg H.V., National Academies of Sciences, Engineering, and Medicine Committee on Examining the Working Definition for Long Covid (2024). Long Covid Defined. N. Engl. J. Med..

[52] Willyard C. (2023). Are repeat COVID infections dangerous? What the science says. Nature..

[53] Liu X., Wu Y., Huo Z., Zhang L., Jing S., Dai Z., Huang Y., Si M., Xin Y., Qu Y. (2024). COVID-19 Vaccine Hesitancy Among People Living with HIV: A Systematic Review and Meta-Analysis. AIDS Behav..

