# Supplementary material for: COVID-19 Vaccination and Transient Increase in CD4/CD8 Cell Counts in People with HIV: Evidence from China
Source: Vaccines (Basel). 2024 Dec 3;12(12):1365. doi: 10.3390/vaccines12121365 (PMC11680300; doi:10.3390/vaccines12121365)
Supplement: Supplementary file 1 [file vaccines-12-01365-s001.zip › vaccines-3290544-supplementary.pdf]

Supplementary Table S1 Pre-COVID-19 biochemical indicators between the matched groups.

Characteristics	Unvaccinated (n = 34)	Vaccinated (n = 96)	P
Liver function indicators			
Alanine aminotransferase, (U/L)	20.9 (16.4, 33.9)	21.2 (16.7, 42.1)	0.513
Aspartate aminotransferase, (U/L)	21.4 (17.5, 33.0)	21.6 (17.2, 26.9)	0.699
Direct bilirubin, (μmol/L)	3.3 (2.8, 4.5)	3.7 (2.6, 5.0)	0.699
Albumin, (g/L)	46.3 ± 6.1	47.3 ± 5.0	0.902
Metabolic parameters, (mmoI/L)			
Total cholesterol	3.9 (3.4, 4.8)	4.0 (3.5, 4.7)	0.973
Triglyceride	1.2 (0.8, 1.7)	1.1 (0.8,1.9)	0.853
Low-density lipoprotein cholesterol	2.5 (2.0, 2.9)	2.4 (2.0, 2.9)	0.590
Renal function parameter			
eGFR, (ml/min/1.73m^2)	116.6 ± 16.0	115.9 ± 14.8	0.821
Creatinine, (μmol/L)	70.3 ± 12.8	71.6 ± 12.7	0.609
Blood routine variables			
White blood cell, (10^9/L)	5.9 ± 2.4	6.0 ± 2.1	0.861
Hemoglobin, (g/L)	137.9 ± 27.9	140.4 ± 17.1	0.273
Platelet, (10^9/L)	222.9 ± 71.0	218.3 ± 70.7	0.745
Inflammatory indicator			
C-reactive protein, (mg/L)	1.7 (0.4, 9.4)	1.2 (0.6, 3.7)	0.405

Abbreviations: Values are median (interquartile range), number (percentage) or mean ± standard deviation. COVID-19, coronavirus disease 2019; eGFR, estimated glomerular filtration rate.

Supplementary Table S2 Comparison of CD4/CD8 counts between post-vaccination and post-COVID-19.

Characteristics	Post-vaccination	Post-COVID-19	P
After first dose (n = 48)			
CD4 count, cells/uL	586.0 (461.5, 718.0)	754.0 (482.0, 895.0)	0.091
CD4/CD8 ratio	0.82 (0.68, 0.96)	0.87 (0.61, 1.05)	0.669
After second dose (n = 124)			
CD4 count, cells/uL	618.0 (452.0, 744.0)	682.0 (482.0, 803.0)	0.065
CD4/CD8 ratio	0.71 (0.53, 0.96)	0.79 (0.59, 0. 97)	0.316
After third dose (n = 122)			
CD4 count, cells/uL	577.5 (440.8, 754.8)	632.0 (486.0, 632.0)	0.089
CD4/CD8 ratio	0.79 (0.53, 1.00)	0.80 (0.55, 1.00)	0.509

Abbreviations: Values are median (interquartile range). COVID-19, coronavirus disease 2019.

Supplementary Table S3 Baseline information on PWH receiving different vaccination doses.

Characteristics	Total (n = 337)	Vaccine doses received			
		One dose (n = 11)	Two doses (n = 77)	Three doses (n = 249)	P
Personal history					
Age, (years)	31.3 ± 8.4	34.0 ± 11.9	30.9 ± 8.3	31.3 ± 8.3	0.511
Male, n (%)	324 (96.1)	10 (90.9)	75 (96.1)	240 (96.4)	0.523
BMI, (kg/m^2)	23.7 ± 2.3	22.7 ± 1.3	23.7 ± 2.5	23.8 ± 2.3	0.305
Smoking, n (%)	142 (42.1)	6 (54.5)	31 (40.3)	105 (42.2)	0.786
Drinking, n (%)	119 (35.3)	6 (54.5)	21 (27.3)	92 (36.9)	0.67
Comorbidities, n (%)					
Hypertension	14 (4.2)	2 (18.2)	2 (2.6)	10 (4.0)	0.165
Diabetes mellitus	25 (7.4)	2 (18.2)	2 (2.6)	21 (8.4)	0.077
Cardiovascular disease	5 (1.5)	1 (9.1)	1 (1.3)	3 (1.2)	0.336
Chronic kidney disease	32 (9.5)	3 (27.3)	7 (21.9)	22 (8.8)	0.226
HIV markers					
CD4 count, cells/uL					
Nadir	349.0 (200, 498.5)	233.0 (165.5, 507.0)	324.0 (181.0, 493.0)	359.0 (204.0, 498.0)	0.381
Pre-COVID-19	562.0 (401.5, 741.0)	434.0 (302.5, 637.5)	536.0 (373.0, 733.0)	580.0 (436.0, 740.0)	0.132
CD8 count, cells/uL					
Nadir	880.0 (612, 1225.5)	712.0 (559.5, 1259.5)	785.0 (572.0, 1159.0)	930.0 (634.0, 1248.0)	0.313
Pre-COVID-19	862.0 (630.0, 1160.5)	607.0 (516.0, 898.0)	826.0 (608.0, 1099.0)	889.0 (686.0, 1192.0)	0.062
CD4/CD8 ratio					
Nadir	0.35 (0.21, 0.57)	0.29 (0.16, 0.38)	0.34 (0.22, 0.54)	0.36 (0.21, 0.59)	0.305
Pre-COVID-19	0.65 (0.47, 0.89)	0.67 (0.49, 0.85)	0.65 (0.46, 0.80)	0.65 (0.48, 0.92)	0.879
Time of ART initiation, years	4.0 (1.3,6.0)	2.3 (0.9, 5.0)	3.5 (0.6, 6.0)	4.0 (1.5, 6.0)	0.487

Abbreviations: Values are mean ± standard deviation, number (percentage) or median (interquartile range). PWH, people with HIV; BMI, body mass index; COVID-19, coronavirus disease 2019; ART, antiretroviral therapy.

Supplementary Table S4 HIV markers in PWH receiving different vaccination doses.

Characteristics	Total (n = 337)	Vaccine doses received		
		One dose (n = 11)	Two doses (n = 77)	Three doses (n = 249)
Pre-pandemic				
CD4 count, cells/uL	562.0 (401.5, 741.0)	434.0 (302.5, 637.5)	536.0 (373.0, 733.0)	580.0 (436.0, 740.0)
CD8 count, cells/uL	862.0 (631.0, 1160.5)	672.0 (580.0, 898.0)	826.0 (608.0, 1099.0)	889.0 (686.0, 1192.0)
CD4/CD8 ratio	0.65 (0.47, 0.89)	0.67 (0.49, 0.85)	0.65 (0.46, 0.80)	0.65 (0.48, 0.92)
Post-COVID-19				
CD4 count, cells/uL	669.0 (456.0, 861.5)	546.0 (441.5, 808.5)	583.0 (414.0, 833.0)	685.0 (494.0, 877.0)
CD8 count, cells/uL	828.0 (617.0, 1110.5)	733.0 (658.0, 990.5)	796.0 (565.0, 1241.0)	851.0 (640.0, 1085.0)
CD4/CD8 ratio	0.80 (0.60, 1.00)	0.70 (0.65, 0.90)	0.70 (0.60, 1.00)	0.80 (0.60, 1.00)
Recent				
CD4 count, cells/uL	644.0 (478.5, 813.0)	663.0 (431.5, 739.5)	601.0 (427.0, 826.0)	656.0 (493.0, 810.0)
CD8 count, cells/uL	788.0 (0.59, 1.08)	727.0 (553.0, 775.0)	792.0 (582.0, 1084.0)	798.0 (595.0, 1068.0)
CD4/CD8 ratio	0.78 (0.59, 1.08)	0.95 (0.78, 1.04)	0.72 (0.56, 1.13)	0.81 (0.62, 1.07)

Abbreviations: Values are median (interquartile range). PWH, people with HIV.

Supplementary Table S5 Changes in biochemical indicators in PWH with previous COVID-19 infection.

Characteristics	Pre-COVID-19 (n = 328)	Recent (n = 328)	P
Alanine aminotransferase , (U/L)	26.0 (18.9, 40.1)	25.4 (18.7, 38.9)	0.134
Aspartate aminotransferase, (U/L)	21.8 (17.7, 27.2)	20.4 (16.7, 25.9)	0.081
Albumin, (g/L)	48.1(46.3, 49.7)	47.3 (46.0, 49.0)	0.006
eGFR, (ml/min/1.73m^2)	116.5 (108.3, 120.9)	107.7 (93.5, 115.4)	<0.001
Glucose, (mmol/L)	5.6 (5.3, 6.0)	5.5 (5.1, 5.9)	0.062
Triglyceride, (mmoI/L)	4.3 (3.7, 4.8)	4.4 (3.9, 5.0)	0.109
Total cholesterol, (mmoI/L)	1.4 (0.9, 2.1)	1.3 (0.9, 2.0)	0.510
C-reactive protein, (mg/L)	1.0 (0.5, 2.3)	1.3 (0.6, 2.3)	0.139

Abbreviations: Values are median (interquartile range). PWH, people with HIV; eGFR, estimated glomerular filtration rate.
